# Air-Drying of Cells, the Novel Conditions for Stimulated Synthesis of Triacylglycerol in a Green Alga, *Chlorella kessleri*

**DOI:** 10.1371/journal.pone.0079630

**Published:** 2013-11-15

**Authors:** Takuma Shiratake, Atsushi Sato, Ayumi Minoda, Mikio Tsuzuki, Norihiro Sato

**Affiliations:** 1 School of Life Sciences, Tokyo University of Pharmacy and Life Sciences, Hachioji, Tokyo, Japan; 2 JST, CREST, Chiyoda-ku, Tokyo, Japan; National Institute for Viral Disease Control and Prevention, CDC, China

## Abstract

Triacylglycerol is used for the production of commodities including food oils and biodiesel fuel. Microalgae can accumulate triacylglycerol under adverse environmental conditions such as nitrogen-starvation. This study explored the possibility of air-drying of green algal cells as a novel and simple protocol for enhancement of their triacylglycerol content. *Chlorella kessleri* cells were fixed on the surface of a glass fibre filter and then subjected to air-drying with light illumination. The dry cell weight, on a filter, increased by 2.7-fold in 96 h, the corresponding chlorophyll content ranging from 1.0 to 1.3-fold the initial one. Concomitantly, the triacylglycerol content remarkably increased to 70.3 mole% of fatty acids and 15.9% (w/w), relative to total fatty acids and dry cell weight, respectively, like in cells starved of nitrogen. Reduction of the stress of air-drying by placing the glass filter on a filter paper soaked in H_2_O lowered the fatty acid content of triacylglycerol to 26.4 mole% as to total fatty acids. Moreover, replacement of the H_2_O with culture medium further decreased the fatty acid content of triacylglycerol to 12.2 mole%. It thus seemed that severe dehydration is required for full induction of triacylglycerol synthesis, and that nutritional depletion as well as dehydration are crucial environmental factors. Meanwhile, air-drying of *Chlamydomonas reinhardtii* cells increased the triacylglycerol content to only 37.9 mole% of fatty acids and 4.8% (w/w), relative to total fatty acids and dry cell weight, respectively, and a marked decrease in the chlorophyll content, on a filter, of 33%. Air-drying thus has an impact on triacylglycerol synthesis in *C. reinhardtii* also, however, the effect is considerably limited, owing probably to instability of the photosynthetic machinery. This air-drying protocol could be useful for the development of a system for industrial production of triacylglycerol with appropriate selection of the algal species.

## Introduction

Triacylglycerol (TG), which has three esterified fatty acid molecules on its glycerol backbone, is ubiquitous in eukaryotes, and is also present in some groups of prokaryotes [1]. Generally, TG is deposited in cells, and more specifically in an organelle designated as lipid droplets, which are surrounded by a monolayer of polar lipids. Also included in the lipid droplets are proteins that determine the size and stability of the lipid droplets, and enzymes concerning the metabolism of neutral lipids including TG [2]. Fatty acids can be released from TG in lipid droplets on demand as a carbon and/or energy source, e.g., when the seeds of terrestrial plants germinate [3], and thus can be regarded as being crucial as a storage compound.

Industrially, TG is utilized as a starting material for a variety of commercial products. Above all, the production of biodiesel from plant-derived TG has recently attracted intense attention [4], due to the increasing necessity for renewable energy sources to cope with the global elevation in the level of atmospheric carbon dioxide, and also as a substitute for fossil fuels, which will become exhausted in the future. At present, plants cultivated for biodiesel production include food crops such as soybean and rapeseed [The European Biodiesel Board (http://www.biodiesel.org)], which consequently could cause competition between biodiesel and food production. Meanwhile, TG purified from oil palm is utilized widely for the production of commodities such as food oil and soap. However, there has been the problem for more than three decades of the destruction of the tropical rainforest in Indonesia due to the continuing development of large-scale oil palm plantations [5]. Algae are attractive for overcoming these problems since they are not usually consumed as human food and can be cultivated even on barren land if there are suitable culturing facilities. Furthermore, algal cells can be cultured throughout the year, which enables an annual biomass yield exceeding that produced by terrestrial plants by ca. 10-fold [4].

In this context, it is of note that a variety of algal species exhibit increases in the cellular content of neutral lipids, TG in particular, when they suffer from environmental stresses such as nutritional starvation, high salinity, and intense light, or when they enter the stationary phase during growth [4]. Oleaginous algal species that can have lipid contents of excess of 20% dry mass have been found in diverse taxonomic groups including green and red algae, and secondary symbiotic algae such as diatoms [4]. Nitrogen (N)-deficiency is a representative stress that promotes lipid synthesis in algal species, e.g., *Nannochloropsis* sp. QII, which is a Eustimatophyte, such that the cellular lipid content increases to 55% of its biomass owing mainly to marked accumulation of TG to 79% of the total lipid content [6]. However, it would be economically difficult to apply N-starvation to large scale production of algal TG in view of the slow growth rates in general of such oleaginous algal strains, and the labor and cost for exchange of the culture medium [4].

In this study, we investigated the effects of air-drying of cells on TG accumulation in two green algae, *Chlorella kessleri* and *Chlamydomonas reinhardtii*, with the aim of developing a simple and thus low-cost protocol. Both species show high growth rates, and thus have frequently been used for studying basic scientific aspects such as lipid metabolism, including the synthesis of TG [7], [8]. Moreover, a large-scale culturing system would be possible for *C. kessleri*, in particular, in view of the established system for *Chlorella* sp., which has contributed for decades to the production of fertilizers, fish culture feed, and food additives (e.g., see http://www.chlorella.co.jp/). We here found that air-drying significantly stimulates TG synthesis in cells of these two green algae, especially *C. kessleri*, due to the effects of both dehydration and nutritional depletion. This novel protocol should be utilizable for the development of a system for large-scale production of algal TG.

## Materials and Methods

### Strains and pre-growth conditions

Two green algae, *C. kessleri* 11h [9] and *C. reinhardtii* CC125 [8], were used. The cells were pre-cultured at 30°C with aeration in an oblong glass vessel under illumination (39 µmol photons·m^−2^·s^−1^) until the OD_730_ value became ca. 0.5 and 0.3 for *C. kessleri* and *C. reinhardtii*, respectively. The culture media for *C. kessleri* and *C. reinhardtii* were 4-fold diluted Gamborg's B5 medium [10] and 3/10 HSM [11], respectively. For N-starvation stress, *C. kessleri* cells grown to the linear phase in 3/10 HSM were harvested by centrifugation, washed twice, and then resuspended in N-free 3/10 HSM for further growth for 120 h.

### Protocol for air-drying of cells

**Figure 1:**
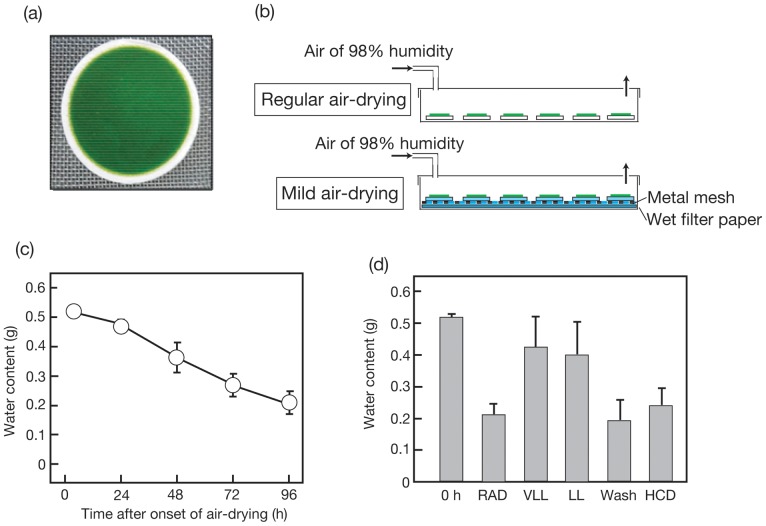
Protocol for air-drying of *C. kessleri* cells and its effects on dehydration of the cells. Cells were layered on a glass microfibre filter through vacuum-filtration (a), and thereafter were placed in a plastic box for aeration at 98% humidity for RAD or mild air-drying as described under Materials and Methods (b). (c) Drying of a glass microfibre filter, on which cells were layered, during RAD. (d) VLL and LL represent light-intensity conditions of 1.6 and 3.9 µmol photons·m^−2^·s^−1^, respectively, during air-drying. Wash represents the use of cells that were washed once with H_2_O before the onset of RAD, whereas HCD indicates that a 3-fold higher density of cells was placed on the glass fibre filter. The values are the averages ± SE for three to four independent cultures. Some SE bars were hidden by symbols, owing to their small values.

To impose regular air-drying stress (hereafter referred to as RAD) on the cells (Fig. 1b), 50 mL of a pre-culture in the early linear growth phase was vacuum-filtered on a glass microfibre filter (47-mm diameter, GF/C. Buckinghamshire, Whatman. Fig. 1a). It was found that the chlorophyll (Chl) content on a filter ranged from 248 to 280 µg for *C. kessleri*, and from 85 to 175 µg for *C. reinhardtii*. Thereafter, the cells were incubated on the filter at 30°C for 96 h in a plastic box with illumination with fluorescent light (15 µmol photons·m^−2^·s^−1^). For slow drying of the filter, the air was circulated with an air pump with as high as 98% humidity, and at a rate that allowed the air in the chamber to be replaced with in one hour. On the other hand, for less severe air-drying (hereafter referred to as mild air-drying), the glass filter on which the cells were layered was put on a metal mesh (mesh size, 1 mm^2^) over a filter paper soaked in culture medium or H_2_O (Fig. 1a, b). For measurement of dry cell weight (DCW), the cells on the glass filter were placed in a dry heat sterilizer (FS-420. Advantec, Tokyo) at 55°C for more than 5 h. Since there was no significant effect of this heat treatment on the weight of a glass fibre filter itself (data not shown), the filter without heat treatment was pre-weighed for calculation of DCW. The H_2_O content of the glass filter was estimated as the difference between the weights of the filter before and after the heat treatment at 55°C.

### Extraction of Chl and lipids, and their quantitative analysis

Chl was extracted from the cells on a glass filter with methanol, and thereafter quantified spectrophotometrically as described in [11]. Total lipids were extracted from the cells on the glass filter according to the method of Bligh and Dyer, as previously described [11], and then separated into individual neutral lipid classes by TLC with a solvent system of hexane/diethyl ether/acetate (70:30:1, by vol.). The lipid spots were visualized by spraying of primuline [11]. From the total lipids and the silica gel corresponding to the TG spot that had been cut out from a TLC plate, fatty acid methyl esters were prepared with 5% anhydrous methanolic HC1 for analysis by capillary GLC with the use of arachidic acid as an internal standard, as described previously [11]. The Chl content, constituent fatty acids of TG, and total fatty acids in cellular lipids (TFA) were, respectively, quantified on a glass filter basis. The content of TG including its glycerol backbone was estimated on the basis of its constituent fatty acids. The fatty acid content of polar lipids was estimated by subtraction of that of TG from TFA, in view of the predominant occupation of neutral lipids by TG (see below).

### Microscopic observation of lipid droplets

A glass filter, on which green algal RAD cells had been placed, was transferred to a tube containing the culture medium, followed by vigorous shaking. A Nile red solution (0.25 mg/ml in acetone) was added to the resultant cell suspension (1:50, v/v), and stained cells were observed under a fluorescence microscope (BX-FLA. Olympus Optical Co., Tokyo, Japan) with the use of a 520–550 nm excitation filter.

## Results

### Accumulation of TG in cells of *C. kessleri* 11h subjected to air-drying stress

**Figure 2:**
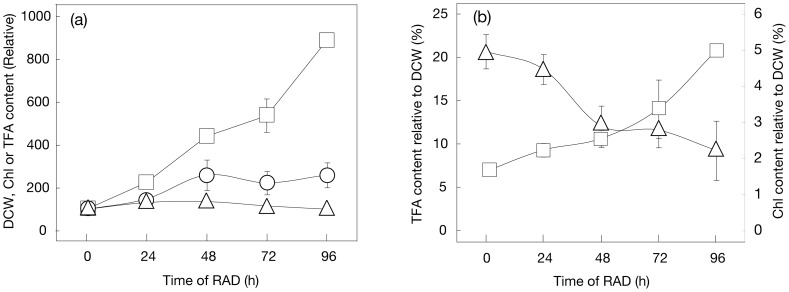
RAD-induced changes in DCW, and Chl or TFA content, in *C. kessleri*. (a) Cells were cultured under RAD conditions, and harvested at the designated times for analysis of DCW (circles), and the Chl (triangles) and TFA (squares) contents on a filter basis. The values shown are relative to the respective initial levels taken as 100%. (b) Contents of Chl (triangles) and TFA (squares) were estimated relative to DCW on the basis of data in (a). The values are the averages ± SE for three independent cultures. Some SE bars were hidden by symbols, owing to their small values.

**Figure 3:**
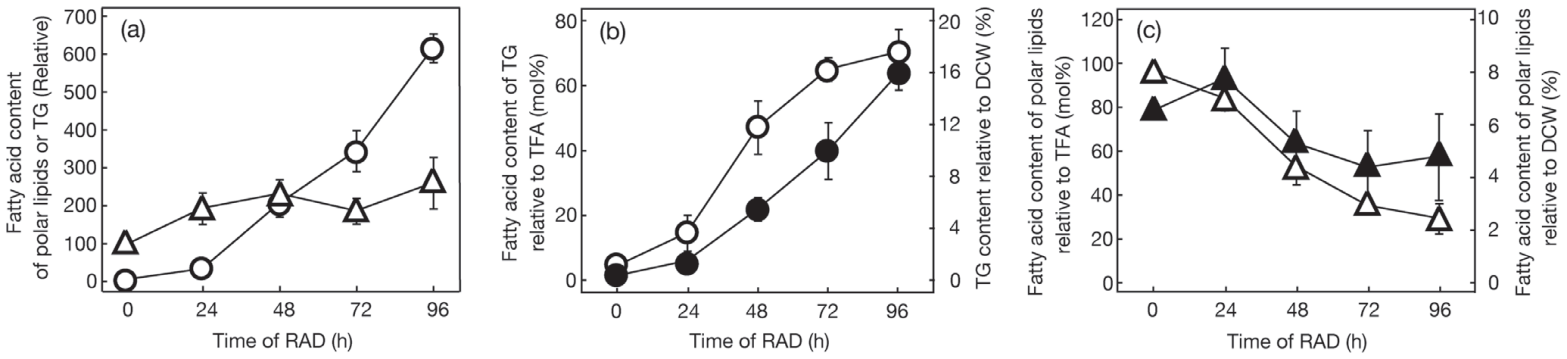
RAD-induced quantitative changes in TG and polar lipids in *C. kessleri*. (a) Cells were cultured under RAD conditions, and harvested at the designated times for analysis of the fatty acid contents of TG (circles) and polar lipids (triangles) on a filter basis. The values shown are relative to the TFA content at 0 h taken as 100%. (b) Contents of fatty acids of TG relative to TFA (open circles) or TG content relative to DCW (closed circles) were estimated. (c) Contents of fatty acids of polar lipids relative to TFA (open triangles) or DCW (closed triangles) were estimated. The values are the averages ± SE for three independent cultures. Some SE bars were hidden by symbols, owing to their small values.

**Figure 4:**
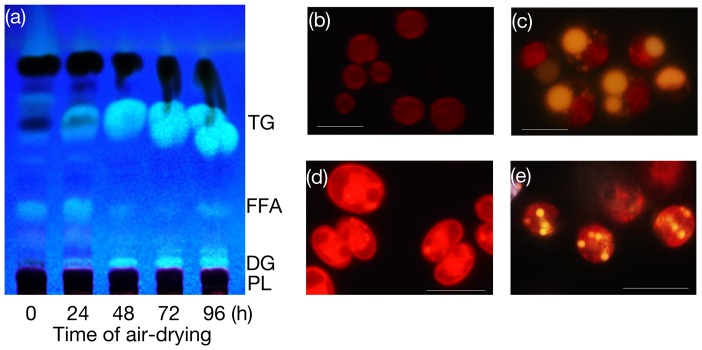
RAD-induced accumulation of TG and lipid droplets in green algae. (a) TLC profile of neutral lipids extracted from RAD cells of *C. kessleri* at the designated times. FFA, free fatty acids. DG, diacylglycerol. PL, polar lipids. Lipid droplets were observed in Nile-Red stained cells under a fluorescence microscope as described under Materials and Methods. *C. kessleri* cells before (b) and after RAD for 96 h (c). *C. reinhardtii* cells before (d) and after RAD for 96 h (e). White bars represent 10 µm.

Several environmental stresses are known to induce marked synthesis of TG in numerous species of eukaryotic algae [4]. Here, we investigated the effect of air-drying on the TG content of cells of a green alga, *C. kessleri*. Cells that had been normally cultured in advance in liquid medium were layered on the surface of a glass microfibre filter (Fig. 1a), and thereafter subjected to RAD for 96 h (Fig. 1b). This treatment should lead the cells to a severe dehydrated state in line with a decrease in the H_2_O content in a glass microfibre filter carrying cells from 0.5 to 0.2 mg in 96 h (Fig. 1c). Despite the unfavorable growth conditions, DCW on a filter basis increased considerably to 2.7-fold of the initial level in 48 h, and then plateaued at this increased level (Fig. 2a). The Chl content of the filter, distinct from DCW, was only 1.3-fold increased at 48 h, thereafter returning to the initial level at 96 h (Fig. 2a). As a result, the Chl content relative to DCW decreased from 5 to 2% (w/w) (Fig. 2b). Meanwhile, the TFA content on a filter basis increased to as much as >8-fold in 96 h (Fig. 2a), which led to an increase in it of 6.8 to 20.8% (w/w) relative to DCW (Fig. 2b). This elevation of TFA on a filter basis was due mainly to a >130-fold increase in the fatty acid content of TG, which occupied a large part of the neutral lipids (Figs. 3a and 4a), and, to a much lesser extent, to a 2-fold one in the fatty acid content of polar lipids (TFA content minus fatty acid content of TG, Fig. 3a). We estimated that the content of TG was elevated from <0.3 to 15.9% (w/w) relative to DCW, or from <4.7 to 70.3 mole% of fatty acids relative to TFA (Fig. 3b). This accumulation of TG was compatible with the finding on observation of Nile-red stained RAD cells under a fluorescence microscope that lipid droplets appeared as a few large intracellular globules that emitted yellow fluorescence against the background of red autofluorescence of Chl (Fig. 4c, cf. Fig. 4b). In contrast, the fatty acid content of total polar lipids decreased from >6.5 to 4.9% (w/w) relative to DCW, or from >95.3 to 29.7 mole% as to TFA (Fig. 3c). Since TG accumulated to occupy 72.9±8.7 mole% TFA in cells of *C. kessleri* grown for four days in liquid medium deprived of an N-source, it was found that RAD stress is as effective as N-deficiency stress for induction of TG accumulation.

**Figure 5:**
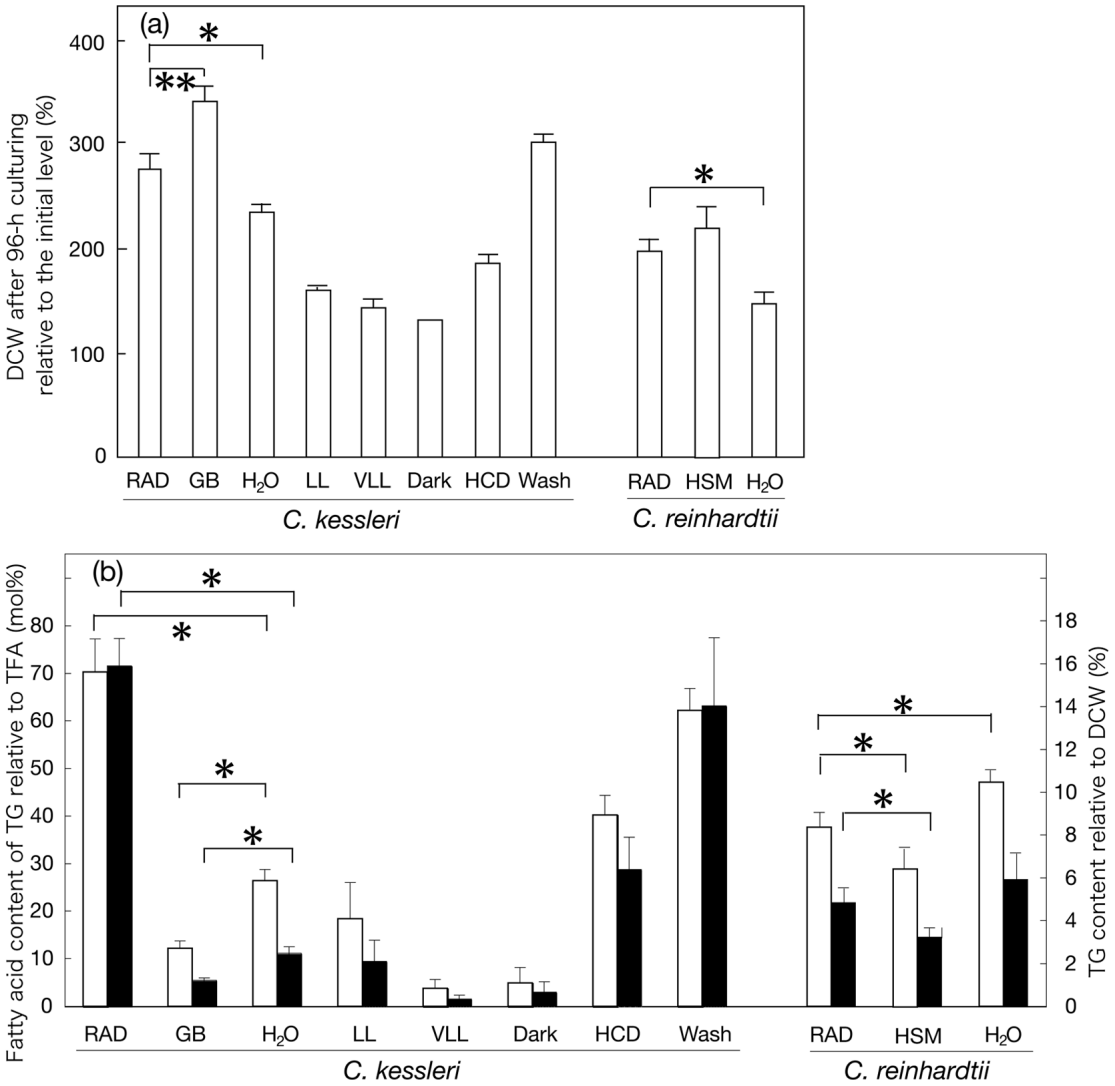
Environmental factors responsible for RAD-stimulated synthesis of TG in *C. kessleri* and their validity in *C. reinhardtii*. DCW on a filter basis (a) and the fatty acid content of TG relative to TFA (open bars) or TG content relative to DCW (closed bars) (b) are analyzed after 96-h culturing under various conditions in *C. kessleri* or *C. reinhardtii*. GB, HSM, and H_2_O indicate mild air-drying conditions with a supply of 4-fold diluted Gamborg’s B5 medium, 3/10 HSM, and H_2_O, respectively. VLL and LL represent low light-intensity conditions during RAD stress and HCD indicates a high cell density placed on a glass filter, as explained in Fig. 1. Wash represents the use of cells that were washed once with H_2_O before the onset of RAD. The values are the averages ± SE for three to five independent cultures, except those of ‘Dark’ in (a), which represent the averages for two independent cultures. The significance of differences was evaluated by Student’s t test. *, P<0.05. **, P<0.1.

RAD causes not only dehydration stress, but also decreased availability of nutritional salts. We then explored environmental factor(s) that are crucial for the substantial accumulation of TG in RAD cells (Fig. 5). Cells subjected to mild air-drying stress with a supply of H_2_O (Fig. 1b) showed an increase in DCW on a filter basis of 2.2-fold of the initial level in 96 h, and thus showed retarded growth compared to RAD cells (Fig. 5a, H_2_O, cf. RAD). Simultaneously, the accumulation of TG only to 26.4 mole% of fatty acids as to TFA and 2.5% (w/w) relative to DCW was observed, i.e. the values were much lower than those observed for RAD cells, indicating that severe dehydration caused by RAD stress is required for full induction of TG synthesis (Fig. 5b, H_2_O, cf. RAD). Meanwhile, replacement of the H_2_O with the culture medium during the mild air-drying resulted in more vigorous growth such that DCW on a filter basis increased to 3.4-fold of the initial level (Fig. 5a, GB, cf. H_2_O). Notably, this nutritional enrichment caused even slower elevation of the TG content to only 12.2 mole% of fatty acids and 1.2% (w/w) relative to TFA and DCW, respectively (Fig. 5b, GB cf. H_2_O). It thus was likely that nutritional depletion is another key factor for full induction of TG synthesis in RAD cells. Overall, it can be supposed that critical environmental factors for TG accumulation in RAD cells of *C. kessleri* comprise at least dehydration and nutrient deficiency, and that dehydration stress should be severely imposed by RAD for the maximal impact on TG synthesis.

### Practical conditions necessary for TG accumulation in cells of *C. kessleri* during air-drying

We explored the optimal conditions for accumulation of TG in *C. kessleri* cells during air-drying in view of its potential application to the industrial production of TG. First, we examined the effects of dark to low light intensities (Fig. 5), as compared with that of regular light (15 µmol photons·m^−2^·s^−1^). The dark or lowered light intensity conditions resulted in significant repression of the increase in biomass during air-drying for 96 h [only 1.3-, 1.4-, and 1.6-fold increases in DCW for 0 (Dark), 1.6 (very low light, VLL), and 3.9 µmol photons·m^−2^·s^−1^ (low-light, LL), respectively, cf. a 2.7-fold increase for 15 µmol photons·m^−2^·s^−1^ (Fig. 5a)]. Meanwhile, the level of H_2_O retained in the glass filter after air-drying for 96 h was 2-fold higher under the VLL or LL conditions than under the regular light conditions (Fig. 1d). A decrease in the light-intensity from 15 µmol photons·m^−2^·s^−1^ to LL lowered the content of accumulated TG from 70.3 to 18.4 mole% of fatty acids as to TFA or from 15.9 to 2.1% (w/w) relative to DCW (Fig. 5b). The elevation of the TG content caused by RAD was abolished almost completely under the dark or VLL conditions. These results revealed that the RAD-induced production of TG depends on illumination with at least 15 µmol photons·m^−2^·s^−1^ in *C. kessleri* cells. The regular light conditions only slightly elevated the temperature in the chamber (<0.2°C).

The cell density on the glass filter would be another factor that affects the TG productivity. A 3-fold larger population of cells was then fixed on a glass filter for growth for 96 h under RAD conditions, which allowed DCW on a filter basis to increase to only 1.8-fold of the initial level [Fig. 5a, HCD (high cell density), cf. RAD]. HCD brought about an incomplete increase in the TG content to 40.2 mole% of fatty acids or 6.4% (w/w) relative to TFA and DCW, respectively (Fig. 5b). The HCD therefore was estimated to yield only 1.2-fold enhancement of the TG productivity on a glass filter, and thus was found to be of no great advantage.

One of the key environmental factors for marked accumulation of TG in RAD cells is nutritional deficiency (see above). With the RAD protocol, however, some medium was retained around the cells on the glass filter (Fig. 1c), which might mitigate the nutritional deficiency. We then washed the cells with H_2_O to thoroughly remove nutrients, thereafter placing the cells on a glass filter for RAD. The resultant cells, however, exhibited little enhancement of the level of accumulated TG, indicating that a low level of medium remaining around the cells on a glass filter never interferes with the cellular response of stimulation of TG accumulation (Fig. 5b, Wash, cf. RAD).

### Evaluation of the effectiveness of air-drying for accumulation of TG in cells of *C. reinhardtii*

It was then investigated whether or not RAD is effective for stimulation of TG synthesis in another green alga, *C. reinhardtii*. We first examined a change in the content of TG in cells that had been subjected to mild air-drying with a supply of the culture medium or H_2_O. The provision of the culture medium, i.e., imposition of less severe dehydration than in the case of RAD, concomitantly with no serious nutritional deficiency, caused an increase in DCW on a filter basis to 2.2-fold in 96 h (Fig. 5a, GB in *C. reinhardtii*). Concomitantly, the TG content increased to 28.1 mole% of fatty acids and 3.2% (w/w), relative to TFA and DCW, respectively (Fig. 5b, GB in *C. reinhardtii*). Substitution of H_2_O for the culture medium resulted in a smaller increase in DCW to 1.5-fold (Fig. 5a, H_2_O in *C. reinhardtii*), with greater accumulation of TG to 47.5 mole% and 5.9% (w/w) (Fig. 5b, H_2_O in *C. reinhardtii*). These results thus confirmed that in *C. reinhardtii*, as in *C. kessleri*, mild dehydration by itself facilitates the cellular accumulation of TG, and that the impact becomes greater with simultaneous nutritional depletion. It was also of note that the ability to accumulate TG was more outstanding in *C. reinhartdii* than in *C. kessleri*.

**Figure 6:**
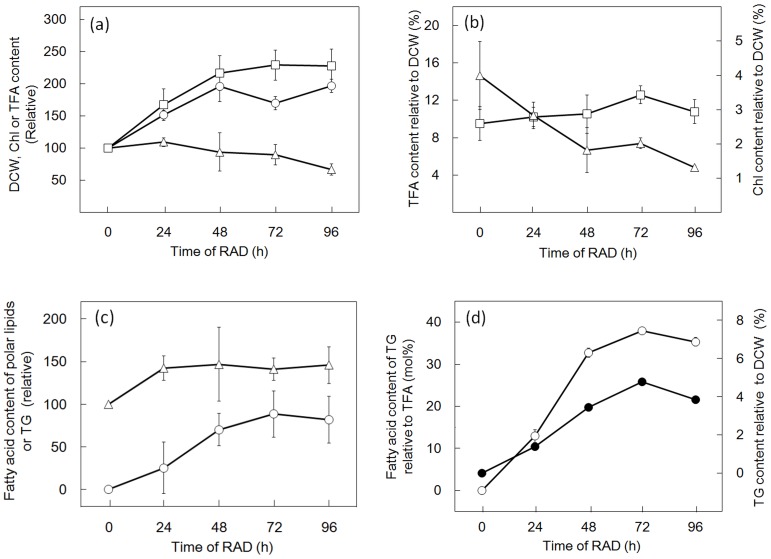
RAD-induced changes in DCW, and Chl, TFA or TG content, in *C. reinhardtii*. (a) Cells were cultured on the glass filter under RAD conditions, and harvested at the designated times for analysis of DCW (circles), and Chl (triangles) and TFA (squares) contents on a filter basis. The values shown are relative to the respective initial levels taken as 100%. (b) Contents of Chl (triangles) and TFA (squares) were estimated relative to DCW on the basis of the data in (a). (c) Contents of fatty acids in TG (circles) and those in polar lipids (triangles) are shown relative to the TFA content at 0 h taken as 100%. (d) Contents of fatty acids of TG relative to TFA (open circles) or TG content relative to DCW (closed circles) were estimated. The values are the averages ± SE for three independent cultures. Some SE bars were hidden by symbols, owing to their small values in RAD cells.

We then examined whether or not the synthesis of TG can be further stimulated in *C. reinhardtii* with dehydration stress due to RAD to surpass that in *C. kessleri* (Figs. 5 and 6). DCW on a filter basis increased in RAD cells of *C. reinhardtii* to 2.0-fold, and thus became higher and lower than in mild air-dried cells supplied with H_2_O and the culture medium, respectively (Fig. 5a), as in *C. kessleri*. This increased DCW ratio, which was smaller than that in *C. kessleri* (2.7-fold, Figs. 2a and 5a), was accompanied by an uncrease in the TFA content on a filter to 2.3-fold at most, and the Chl one considerably decreasing to 66.9% of the initial level (Fig. 6a). The respective changes of TFA and Chl as to quantity on a filter basis were quite distinct from those in *C. kessleri* cells, which exhibited a >8-fold increase in the TFA content with an almost unaltered Chl content (Fig. 2a). As a result, relative to DCW, the TFA content was elevated by only 32% at most, with a drastic reduction in the Chl content to 32.8% of the initial level (Fig. 6b). Eventually, RAD for 96 h caused accumulation of TG to only 37.9 mole% of fatty acids at most, relative to TFA (Figs. 5b and 6d), under circumstances where the fatty acid content of polar lipids was elevated by 1.5-fold (Fig. 6c). Compatible with the results, microscopic observation revealed intracellular lipid droplets of much smaller size in *C. reinhardtii* than in *C. kessleri* (Fig. 4e, cf. Fig. 4d). These results proved that severer dehydration has no advantage in *C. reinhardtii*, and that *C. reinhardtii* is inferior to *C. kessleri* in the TG productivity

## Discussion

### RAD yields novel conditions for algal TG production: simultaneous imposition of dehydration and nutritional depletion stresses

Lipids can be stored in oleaginous algae, occupying 20–75% (w/w) of dry cells, and including TG and alkanes in general, and long-chain hydrocarbons in particular species [12]. The levels of TG accumulated have thus far been reported to increase when algal cells are exposed to some environmental stresses such as N-source deficiency [4]. In this study, RAD was found to be a novel environmental stress for induction of TG synthesis in *C. kessleri*, and, similar to N-starvation, enabled TG to accumulate to as much as 15.9% (w/w) relative to DCW or 70.3 mole% of fatty acids as to TFA (Fig. 3b). The key for stimulation of the synthesis of TG in RAD cells of *C. kessleri* is simultaneous dehydration and decreased availability of nutritional salts (Fig. 5b). Individually, decreased availability of nutrients including N- and S-sources due to RAD should cause reduced global protein synthesis, as reflected in the almost no increase in the Chl content (Fig. 2a), i.e., in the levels of Chl-protein complexes such as the PSI and PSII complexes. It is thus highly probable that this decreased availability of nutritional salts due to RAD in *C. kessleri* led to channeling of a major metabolic flow of carbon, which had been devoted intrinsically to the synthesis of proteins for cell growth, to the synthesis of N- and S-free carbon-storage compounds like TG [7], [13]. On the other hand, dehydration of cells due to RAD should cause turgor loss, which would disorganize membrane systems including membrane proteins, with consequent damage to key physiological functions such as photosynthesis [14]. These impairments, together with those caused by a simultaneous nutritional deficiency, would bring about stimulated production of reactive oxygen species that would further adversely affect physiological processes including protein synthesis, like in plants exposed to drought stress [15], [16]. The addition of 100 mM NaCl to a culture was previously shown to induce TG synthesis in *C. reinhardtii* [13], however, the question of whether the key environmental factor is dehydration caused by high osmotic stress and/or ionic stress has yet to be answered. Dehydration stress seems to be at least one of the factors crucial for the accumulation of TG in salt-stressed cells of *C. reinhardtii* as well as in RAD cells of *C. kessleri*, or in the respective cells of *C. kessleri* and *C. reinhardtii* exposed to mild air-drying with a supply of the culture medium (Fig. 5b). Green algae, like other organisms including yeast and plants, are generally regarded to synthesize TG through the Kennedy pathway, and have often been shown to exhibit up-regulated expression levels of the genes of diacylglycerol acyltransferases that are involved in the terminal step of the pathway, in response to environmental stress conditions that induce accumulation of TG [17]. Elucidation of the molecular mechanism by which the RAD protocol enhances TG synthesis remains for future work.

Besides the marked stimulation of TG synthesis, this novel RAD protocol is attractive in the following two points: one is that there is no need to change the culture medium, distinct from in the case of the nutritional depletion protocol. The other is the curtailing of conventional high-energy input on dewatering of the algal biomass [18], since RAD should remove a much larger amount of culture medium than simple centrifugation of the culture by yielding a thin layer of cells on the glass filter, and subsequent air-drying. These features could thus overcome in part the problems that have so far technologically or economically hampered the industrial algal production of TG. Meanwhile, the ability of *C. reinhardtii* or yeast to accumulate TG has been reinforced through loss of function of a gene for polysaccharide synthesis, lipase, or a regulator of lipid metabolism [13], [19], [20]. *C. kessleri* can be regarded as a microorganism that is close to, but falling short of exhibiting the criteria for oleaginous ones that can accumulate lipids to more than 20% (w/w) DCW, which would be of great industrial interest [21]. It is expected that *C. kessleri*, the cells of which are suitable for chemical or UV mutagenesis, could be genetically modified to enhance their ability to synthesize TG, and that this improvement would lead to their application to the industrial production of TG with the use of RAD. An alternative strategy would be exploration of algal species that are able to tolerate RAD-stress like *C. kessleri* among algal strains known to store more TG than *C. kessleri* [4].

### Role of light in RAD-induced accumulation of TG in *C. kessleri*

Despite the severe environmental stress caused by RAD, DCW significantly increased to 2.7-fold of the initial level in *C. kessleri* cells when they were illuminated with regular light to drive photosynthesis, but not under lowered light-intensity or dark conditions, which implied that photosynthesis led to this increased biomass (Fig. 5a). Accordingly, regular light was required for stimulated accumulation of TG in RAD cells (Fig. 5b), probably for the generation of ATP and NADPH, and carbon-fixed compounds, for fatty acid synthesis. In line with these observations, polar lipids never decreased during RAD-stress, but, on the contrary, increased to 2.0-fold (Fig. 3a), demonstrating the continuing construction of membrane systems in the cells of *C. kessleri*. It thus is likely that the fatty acids required for assembly into TG are ensured at least mainly through their de novo synthesis, rather than through positive degradation of preexisting polar lipids. N-Starved cells of *C. reinhardtii*, similar to RAD ones of *C. kessleri*, ensure such fatty acids for pronounced accumulation of TG predominantly through de novo synthesis [22]. It should also be mentioned that low light, in contrast to regular light, caused only minor dehydration of the glass filter (Fig. 1d). Therefore, apart from a role as a driving force of photosynthesis, light seems to play an additional role in facilitation of dehydration in RAD cells through heat radiation.

Meanwhile, regular light illumination should cause oxidative stress to cells exposed to RAD-stress, as described above. Synthesis of TG, and that of fatty acids in particular, would facilitate the consumption of excessive reducing power that is provided through photosynthesis, thereby preventing the cells from becoming over-reduced in the redox state, and thus from producing radical oxygen species [4]. This regulation of the energy balance would eventually meet the algal requirement for intracellular carbon and energy sources just after the algal cells start to grow again in response to release from the stress conditions.

### Difference in ability of RAD-induced accumulation of TG between *C. kessleri and C. reinhardtii*

As far as the conditions of mild air-drying with either nutritional repletion or depletion are concerned, *C. reinhardtii* exhibited superiority to *C. kessleri* as a biological material for the production of TG (Fig. 5b). It has yet to be determined whether this difference between the two species depends on intrinsic physiological properties of cells of the respective species or on the culture medium used for pre-culturing and/or mild air-drying (3/10 HSM for *C. reinhardtii*, cf. 4-fold diluted Gamborg's B5 medium for *C. kessleri*). However, the TG content in RAD cells of *C. reinhardtii* was lower not only than that in RAD cells of *C. kessleri*, but also than that of mild air-dried cells of *C. reinhardtii* itself. These results definitely demonstrated that RAD cells of *C. reinhardtii* were inferior in TG production to those of *C. kessleri* (Fig. 5b), owing to their sensitivity to severe dehydration stress caused by RAD. Notably, Chl-protein complexes in *C. reinhardtii* are less stable as to RAD than those in *C. kessleri*, since the Chl content was markedly decreased in *C. reinhardtii* (Fig. 6a, cf. Fig. 2a). The pronounced loss of Chl, i.e., that of Chl-protein complexes such as the PSI and PSII complexes, in *C. reinhardtii* should decrease the photosynthetic competence of the cells, which would consequently limit the synthesis of biological materials including TG (Fig. 6a, c, cf. Figs. 2a and 3b).

Morphological analysis has provided evidence that cell walls play a crucial role in acclimation to dehydration stress caused by drought in plants (e.g., [23]-[25]). Two discrete strategies involving the use of cell walls have been proposed for terrestrial green algae, *Klebsormidium* species. Some *Klebsormidium* species protect their cells from water loss with thick and rigid cell walls (> 2 mm), while other ones endure it through proper shrinkage of whole cells with thin and flexible walls, which seems to prevent drastic structural perturbation of organelles [23], [24]. In a seed plant, it is implied, as an acclimation mechanism, that cell walls fold in line with a decrease in cell volume on exposure to drought, which prevents cell membranes being torn away from cell walls [25]. In this context, the sensitivity of *C. reinhardtii* cells to RAD might be correlated to the properties of their walls, and more specifically to the wall components that are occupied dominantly by hydroxyproline-rich glycoproteins [26], distinct from the sugar-based walls in *C. kessleri* cells [27]. In *C. reinhardtii* cells subjected to nutritional depletion of S, the composition of the cell wall proteins changes such that proteins including less abundant S-containing amino acids prevail [26]. It is thus probable that, in RAD cells of *C. reinhardtii*, concomitant deficiencies in S- and N-sources limit the ability to restructure walls, and that the wall system can not cope with severe dehydration stress. Meanwhile, an insoluble and non-hydrolyzable polymer of cell walls designated as algaenan, which green algae such as *Chlorella emersonii* possess, might be required as an H_2_O-impermeable barrier for acquisition of tolerance to air-drying stress [28]. The algaenan contents of *C. kessleri* and *C. reinhardtii* will be compared in the future. In any case, it can be supposed that disturbance of the cell wall system deleteriously affects neighboring cell membranes in *C. reinhardtii*, which is inevitably followed by injury to intracellular functional units such as the photosynthetic machinery. Future study will lead to elucidation of the mechanism underlying the tolerance to dehydration stress in *C. kessleri*, which should open a way to improve the dyhydration-tolerance feature of preferred algal species for utilization for TG production by means of RAD.

## Conclusions

This study showed that RAD yields the novel conditions that markedly induce TG synthesis in a green alga, *C. kessleri*. The key factor is simultaneous imposition of both dehydration and nutritional-deficiency stresses. This protocol could be developed for the industrial large-scale production of TG, with appropriate selection of green algal species that show both high TG-production and RAD-tolerance abilities.
